# IRE-1 endoribonuclease activity declines early in *C. elegans* adulthood and is not rescued by reduced reproduction

**DOI:** 10.3389/fragi.2022.1044556

**Published:** 2022-10-28

**Authors:** Evandro A. De-Souza, Nadia Cummins, Rebecca C. Taylor

**Affiliations:** Neurobiology Division, MRC Laboratory of Molecular Biology, Cambridge, United Kingdom

**Keywords:** UPR, unfolded protein response, proteostasis, cell stress, stress response, *C. elegans*, aging, IRE1

## Abstract

The proteome of a cell helps to define its functional specialization. Most proteins must be translated and properly folded to ensure their biological function, but with aging, animals lose their ability to maintain a correctly folded proteome. This leads to the accumulation of protein aggregates, decreased stress resistance, and the onset of age-related disorders. The unfolded protein response of the endoplasmic reticulum (UPR^ER^) is a central protein quality control mechanism, the function of which is known to decline with age. Here, we show that age-related UPR^ER^ decline in *Caenorhabditis elegans* occurs at the onset of the reproductive period and is caused by a failure in IRE-1 endoribonuclease activities, affecting both the splicing of *xbp-1* mRNA and regulated Ire1 dependent decay (RIDD) activity. Animals with a defect in germline development, previously shown to rescue the transcriptional activity of other stress responses during aging, do not show restored UPR^ER^ activation with age. This underlines the mechanistic difference between age-associated loss of UPR^ER^ activation and that of other stress responses in this system, and uncouples reproductive status from the activity of somatic maintenance pathways. These observations may aid in the development of strategies that aim to overcome the proteostasis decline observed with aging.

## Introduction

A fundamental biological question is why organisms lose their ability to sense and respond to stress as they age. This decline in the function of stress responses leads to a buildup of damaged macromolecules, in particular proteins. Proteins are molecules involved in virtually all the cellular reactions that make life possible, and a network of protein quality control pathways exists to ensure that they maintain their optimal conformation and function. With aging, these quality control processes become less efficient, leading to the accumulation of misfolded proteins and aggregates that are a signature of the onset of neurodegenerative diseases.

A conserved hub in this protein homeostasis (proteostasis) network is the unfolded protein response of the endoplasmic reticulum (UPR^ER^). The UPR^ER^ allows cells to sense and respond to the accumulation of misfolded proteins in the lumen of the endoplasmic reticulum (ER) (Walter and Ron, 2011). In metazoans, the UPR^ER^ can be activated through three distinct branches, controlled by the upstream regulatory molecules IRE1, PERK, and ATF6. The IRE1 branch is the only one conserved from yeast to humans, and plays a role in the aging process and in the pathophysiology of several diseases (Labbadia and Morimoto, 2015b). IRE1 has both kinase and endoribonuclease activities, and responds to proteostatic imbalance through the activation of several downstream mechanisms. At least two of these mechanisms depend upon the endoribonuclease activity of IRE1. These include the splicing of a specific regulated intron from the mRNA of a transcription factor, *XBP1*, which allows spliced *XBP1s* to be translated and to regulate a variety of target genes that promote proteostasis (Calfon et al., 2002). In addition, IRE1 is also able to degrade a variety of ER-localized transcripts through a process called regulated IRE1-dependent decay (RIDD), which reduces the protein folding load in the ER and may also play specific regulatory roles (Hollien and Weissman, 2006; Bae et al., 2019).

The activation of the IRE1 branch of the UPR has been shown to decline with age in the model organism *C. elegans*, as well as in the murine brain and in human cells, but to date, the molecular mechanisms underlying this decline remain elusive (Ben-Zvi et al., 2009; Taylor and Dillin, 2013; Labbadia and Morimoto, 2015a; Cabral-Miranda et al., 2020; Sabath et al., 2020). The identification of the major mechanisms underlying this inhibition might aid in the design of novel interventions to counter age-associated loss of proteostasis and increase human healthspan. It was previously shown that the onset of reproduction affects the ability to transcriptionally activate stress-related genes in *C. elegans* (Labbadia and Morimoto, 2015a). An intriguing hypothesis to explain these results is that upon the onset of the reproductive stage, organisms deviate resources from the soma to maintain the integrity of embryos for the next generation (Maklakov and Immler, 2016). In agreement, animals lacking a germline are more resistant to stress and have extended lifespans (Hsin and Kenyon, 1999; Flatt et al., 2008; Labbadia and Morimoto, 2015a). Activation of the cytosolic heat shock response (HSR) is lost at the onset of reproduction and this loss occurs at the level of chromatin, with increased levels of H3K27me3 histone methylation rendering stress genes inaccessible to the HSR transcription factor HSF-1 (Labbadia and Morimoto, 2015a). This loss of HSR activation can be rescued by mutations that remove the germline stem cells, through rescued expression of the H3K27 demethylase *jmjd-3.1*, levels of which otherwise decline upon the onset of reproduction.

In the same study, ER stress-induced expression of the UPR^ER^ target genes *hsp-3* and *hsp-4* were also found to decline during the first day of adulthood, suggesting that the ability to activate the UPR^ER^ also collapses very early in *C. elegans* aging. In addition, a recent study in a human fibroblast model of aging demonstrated that, in these cells, the ability of the UPR^ER^ transcription factor XBP1 to regulate its target genes declines with senescence while no loss of IRE1-regulated *XBP1* splicing occurs (Sabath et al., 2020). This suggests that the decline in activation of this branch of the UPR^ER^ in senescent human fibroblasts may, like the loss of HSR activity in *C. elegans*, lie at the level of chromatin accessibility. However, it is not clear whether this is also true in non-dividing cells, such as the somatic cells of *C. elegans*, in which age-associated loss of UPR^ER^ activation has previously appeared to lie at the level of *xbp-1* splicing (Taylor and Dillin, 2013). In addition, it is not known whether loss of reproductive capacity can delay this loss of UPR^ER^ activation in the worm, as it does the contemporaneous loss of HSR activation.

We therefore set out to ask when and how the loss of IRE-1/XBP-1 pathway activation occurs in *C. elegans*. We also aimed to discover whether reduced germline function could rescue this decline. Our findings confirm that, as previously suggested, stress-induced UPR^ER^ activation declines within the first day of *C. elegans* adulthood. We also found that loss of IRE-1/XBP-1 pathway function occurs at the level of IRE-1 activation in this organism, upstream of any changes to chromatin accessibility. Finally, to our surprise we found that, unlike the HSR, age-associated loss of UPR^ER^ activation cannot be rescued by a mutation that prevents the formation of the germline. Together, this suggests that the aging-related loss of activation of the UPR^ER^, while occurring at the same time as loss of HSR activation, may happen through a fundamentally different mechanism. This may have important implications for the development of methods to restore UPR^ER^ activation in older cells, in order to develop therapies that target the onset of diseases of aging.

## Methods

### *Caenorhabditis elegans* maintenance

Worms were kept on NGM plates seeded with OP50-1 *E. coli* using standard procedures (Brenner, 1974; Imanikia et al., 2019a; Imanikia et al., 2019b). For assays with the *glp-1*(*e2141*) strain, to induce sterility, worms were synchronized and kept for 48 h at 25°C, and then moved back to 20°C. A list of the strains used in this work can be found in Supplementary Table S1.

### Epifluorescence microscopy

Worm images were obtained using a Leica M205 FA microscope. Worms were immobilized with 10 mM of sodium azide (Sigma Aldrich) before imaging. Fluorescence of worms was quantified using ImageJ.

### RNAi assays

Unless otherwise stated, all RNAi clones used were from the Ahringer RNAi library (Kamath et al., 2003) and were sequenced before use. Bacteria containing RNAi vector were grown overnight in LB with 100 μg/ml carbenicillin (Formedium) and used for seeding NGM plates containing 1 mM IPTG (Generon) and 100 μg/ml of carbenicillin.

### Western blot

Worms were lysed at the beginning of day 1 or day 2 of adulthood and samples normalized by protein content as measured by BCA assay. 30 μg of protein was loaded per lane for SDS-PAGE and gels were transferred using the iBlot system (Invitrogen) before probing with *α*-FLAG (Sigma Aldrich) or *α*-tubulin (Sigma Aldrich) followed by *α*-mouse-HRP (Abcam). Quantification of bands was carried out using ImageJ.

### qRT-PCR

Approximately 150 worms were collected per sample, and RNA extracted. Briefly, a 1:1 mixture of worms and glass beads was added together with a 3x volume of Trizol LS (Life Technologies). The sample was then quickly frozen in liquid nitrogen. Each sample was centrifuged (13,000 g for 1 min), supernatant collected, and an equal volume of ethanol added to the sample. Samples were then processed with a Direct-zol RNA Miniprep kit (Zymo Research). 1 μg of RNA was used for cDNA synthesis using the QuantiTect reverse transcription kit (QIAGEN). The qPCR run was performed in a Vii7 Real-Time PCR machine (ThermoFisher Scientific) and the data was analyzed using the comparative 2ΔΔCt method (Livak and Schmittgen, 2001). The sequence of the primers used in this work can be found in Supplementary Table S2.

### *xbp-1* splicing assay by RT-PCR

Worms were treated with 50 ng/μl of tunicamycin for 4 h, RNA extracted, and cDNA prepared as described above. 2 μl of cDNA was used in a PCR reaction to amplify *xbp-1* spliced and unspliced products. Samples were run on a 2.5% agarose gel stained with SYBR Safe. Quantification of the percentage of spliced *xbp-1* bands was done using ImageJ. The primer sequences used are listed in Supplementary Table S2.

### Statistical analysis

All experiments were performed at least three times. Bars represent the mean and error bars represent the SEM (standard error of the mean). For statistical analysis between two groups, unpaired Student’s t test or Mann-Whitney *U* test were used. For comparisons with more than two groups, One-Way ANOVA with Sidak’s or Tukey’s multiple comparisons tests was used.

## Results

**FIGURE 1:**
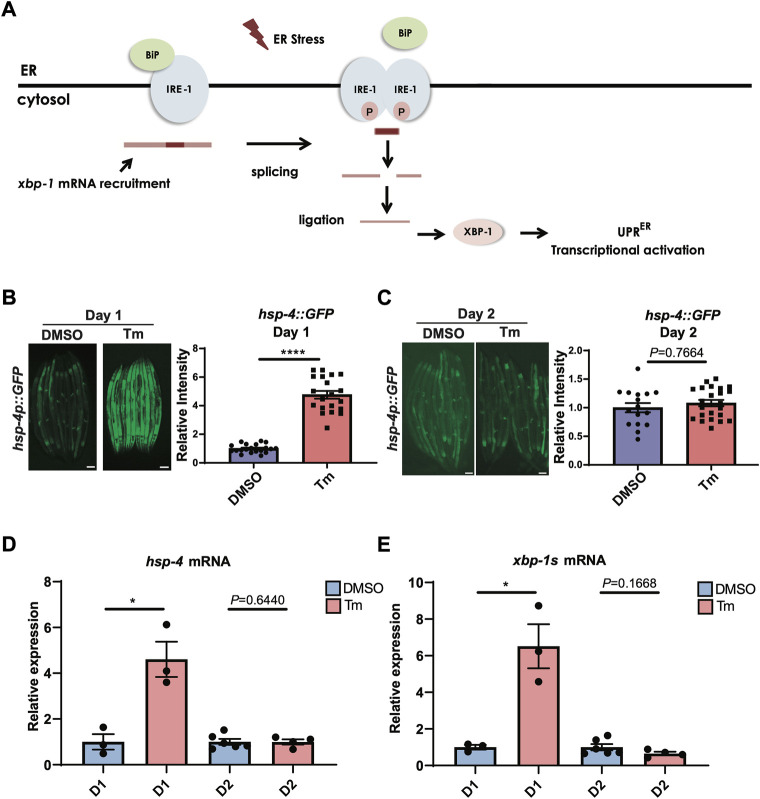
UPR^ER^ activation is lost during the first day of adulthood as animals enter the reproductive period. **(A)** Schematic representation of the IRE-1/XBP-1 branch of the UPR^ER^ in *C. elegans*. IRE-1 is a transmembrane resident ER protein that acts as a sensor of ER stress and transmits that information in a retrograde way to the nucleus. During stress, IRE-1 is autophosphorylated, dimerizes, and together with the RNA ligase RTCB-1 mediates the splicing of the *xbp-1* mRNA in the cytosol, which is translated into an active transcriptional factor. XBP-1 regulates genes such as chaperones that act to alleviate proteotoxic stress in the ER. **(B,C)** Epifluorescence microscopy and quantification of the fluorescence of animals at the beginning of **(B)** Day 1 (D1) or **(C)** Day 2 (D2) of adulthood after 6 h of treatment with tunicamycin (Tm) or DMSO. Scalebars, 200 μm ****p < 0.0001 (Unpaired *t* test with Welch’s correction). **(D,E)** qRT-PCR measurement of **(D)** *hsp-4* and **(E)** *xbp-1s* mRNA after 6 h of tunicamycin (Tm) or DMSO treatment. *p < 0.05, Unpaired *t*-test (DMSO vs. Tm).

Activation of the IRE-1/XBP-1 branch of the UPR^ER^ (Figure 1A) is known to decline with age, but the exact timing of this decline is unclear. As the ability to activate the HSR has been shown to decrease very early in *C. elegans* adulthood, around the onset of the reproductive period (Labbadia and Morimoto, 2015a), we asked whether the same was true of UPR^ER^ activation. Animals expressing an *hsp-4::GFP* UPR^ER^ reporter transgene were treated with the N-linked glycosylation inhibitor tunicamycin, which induces ER stress, at the beginning of the first day of adulthood before egg laying had begun, or at the beginning of day 2 of adulthood. Specifically, animals treated at day 1 of adulthood were within the first 8 h post-L4 larval stage, with oocytes visible within the reproductive system but before the initiation of egg laying, while day 2 animals were 24 h older and actively laying eggs. Following tunicamycin treatment, fluorescence was assessed. In early day 1 of adulthood, UPR^ER^ activation was highly significant, whereas by day 2 it was no longer observable (Figures 1B,C). This suggests that, like the HSR, the ability to activate the UPR^ER^ is lost with the onset of reproduction. To confirm this finding, we also measured transcript levels of the XBP-1 target gene *hsp-4* and levels of the spliced, active form of *xbp-1*, *xbp-1s*, at day 1 and day 2 of adulthood (as above) and found that, again, upregulation upon tunicamycin treatment at day 1 was lost by day 2 (Figures 1D,E).

To ask whether age-associated loss of UPR^ER^ activation was specific to tunicamycin treatment, we also used genetic tools to activate the UPR^ER^. Exposure of *hsp-4p::GFP* animals to RNAi against *pdi-2*, *dnj-7* or *sams-1* robustly activated the UPR^ER^ within 72 h when transfer to plates of RNAi-expressing bacteria occurred at the L1 stage of larval development (Supplementary Figure S1). Some activation was also seen upon transfer at the L3-L4 larval stage. However, transfer at adulthood proved too late to induce UPR^ER^ activation, suggesting that age-associated loss of UPR^ER^ activation is not specific to ER stress-inducing drug treatment. Finally, we asked whether UPR^ER^ activation by heat stress also declined with age. To our surprise, we found that the *hsp-4p::GFP* transgene was still robustly activated by heat stress at day 2 of adulthood (Supplementary Figure S2A). However, UPR^ER^ activation by heat stress proved to be independent of IRE-1 and XBP-1, suggesting that heat stress-induced *hsp-4p::GFP* activation is mediated through an alternative mechanism, and that only *hsp-4p::GFP* activation dependent upon IRE-1 and XBP-1 undergoes age-dependent decline at the onset of reproduction (Supplementary Figure S2B).

**FIGURE 2:**
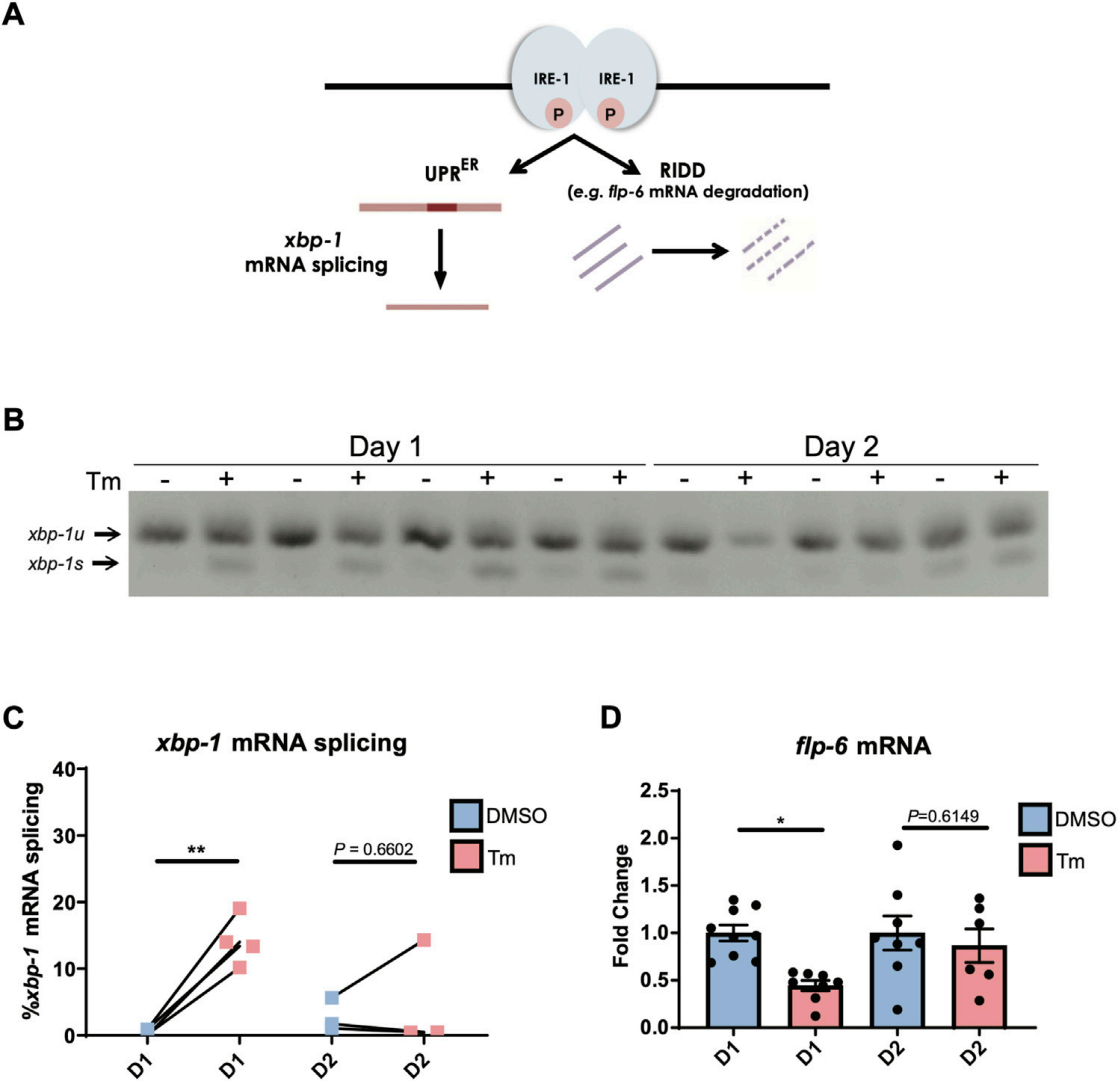
IRE-1 endoribonuclease activities are downregulated during the first day of adulthood as animals enter the reproductive period. **(A)** Schematic representation of IRE-1 endoribonuclease action during ER stress. **(B,C)** *xbp-1* mRNA splicing was measured by qRT-PCR in animals after 6 h of tunicamycin (Tm) or DMSO treatment. **p < 0.01. Unpaired *t*-test (DMSO vs. Tm) **(D)** The mRNA levels of *flp-6*—a RIDD target in *C. elegans*—were measured by qRT-PCR in animals at Day 1 or Day 2 of adulthood after being treated with tunicamycin (Tm) or DMSO for 6 h *p < 0.05, Unpaired *t*-test (DMSO vs. Tm).

Aged worms constitutively expressing a spliced version of *xbp-1* are also capable of inducing *hsp-4p::gfp* past the age at which UPR^ER^ activation usually declines (Taylor and Dillin, 2013), suggesting that loss of UPR activation lies upstream of the transcriptional accessibility of target genes. The absence of spliced *xbp-1s* transcript following tunicamycin-induced UPR^ER^ activation at day 2 of adulthood (Figure 1E) suggests that it is the ability of IRE-1 to splice *xbp-1* mRNA, a core function of this enzyme (Figure 2A), that is lost early in adulthood. We confirmed that *xbp-1* splicing is lost at day 2 of adulthood, while unspliced *xbp-1* is still readily detectable (Figures 2B,C). Given that *xbp-*1 is still transcribed and therefore available for splicing, one explanation for this is that the endoribonuclease function of IRE-1 can no longer be activated at this age. However, it is also possible that *xbp-1* mRNA is no longer recruited to the ER once reproduction has begun, and is therefore not accessible to IRE-1. To distinguish between these possibilities, we asked whether the endoribonuclease-dependent ability of IRE-1 to degrade other ER-localized RNAs, a process known as regulated Ire1-dependent decay (RIDD) (Hollien and Weissman, 2006) (Figure 2A), is also lost by day 2 of adulthood. There is only one confirmed RIDD substrate in *C. elegans*, the neuropeptide *flp-6* (Levi-Ferber et al., 2021). We asked whether *flp-6* mRNA levels still decline upon ER stress induction at day 2 of adulthood, and found that this was no longer the case (Figure 2D). We also asked whether the *C. elegans* homologue of the mammalian RIDD substrate Blos1, *blos-1*, was degraded upon tunicamycin treatment, but found no evidence of reduced *blos-1* transcript levels following tunicamycin exposure (Supplementary Figure S3) (Bae et al., 2019). The loss of *flp-6* degradation coupled with the loss of *xbp-1* splicing therefore suggests that both of the endoribonuclease functions of IRE-1 decline upon the onset of reproduction, lending weight to the possibility that IRE-1 itself fails to become active upon ER stress as cells age.

**FIGURE 3:**
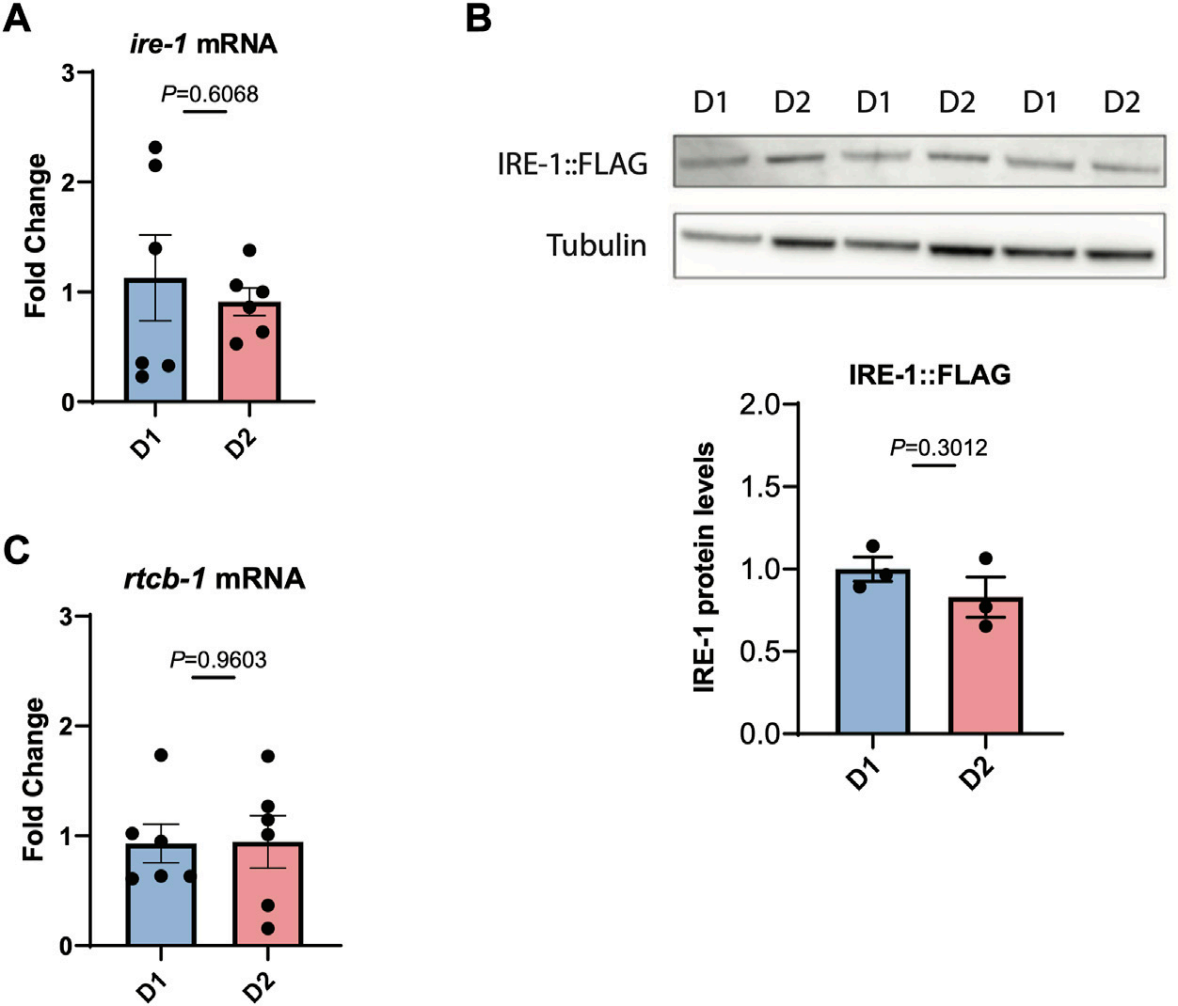
IRE-1 levels are not decreased during the first day of adulthood as animals enter the reproductive period. **(A)** The levels of *ire-1* mRNA were measured by qRT-PCR in animals at the beginning of Day 1 (D1) and Day 2 (D2) of adulthood. **(B)** The levels of endogenously tagged IRE-1-FLAG were monitored in animals by Western blotting with an *α*-FLAG antibody at the beginning of Day 1 (D1) and Day 2 (D2) of adulthood. Band intensities were quantified using ImageJ and normalized to tubulin control bands. **(C)** The levels of *rtcb-1* mRNA were measured by qRT-PCR in animals at the beginning of Day 1 (D1) and Day 2 (D2) of adulthood. Significance in each panel was assessed by Unpaired *t*-test.

We then asked whether this loss of IRE-1 activity might be due to reduced transcription of IRE-1, but we found that *ire-1* transcript levels do not decrease between day 1 and day 2 of adulthood (Figure 3A). We also wondered whether levels of IRE-1 protein might decline with age. To investigate this, we used CRISPR-Cas9 to insert a 3xFLAG tag onto the N-terminus of IRE-1, and used Western blotting with *α*-FLAG to determine endogenous protein levels. We found no decrease in IRE-1 protein levels between day 1 and day 2 of adulthood that would explain the loss of IRE-1 activity between these ages (Figure 3B). Finally, we wondered whether reduced levels of spliced *xbp-1* might result from a failure to re-ligate the *xbp-1* mRNA following splicing by IRE-1, leading to degradation. However, we saw no reduction in transcription of the *xbp-1* ligase *rtcb-1*, suggesting that the loss of spliced *xbp-1* at day 2 of adulthood cannot be explained by reduced expression of this enzyme (Figure 3C). These data therefore suggest that IRE-1 protein is present in aged animals but can no longer mediate its endoribonuclease functions.

**FIGURE 4:**
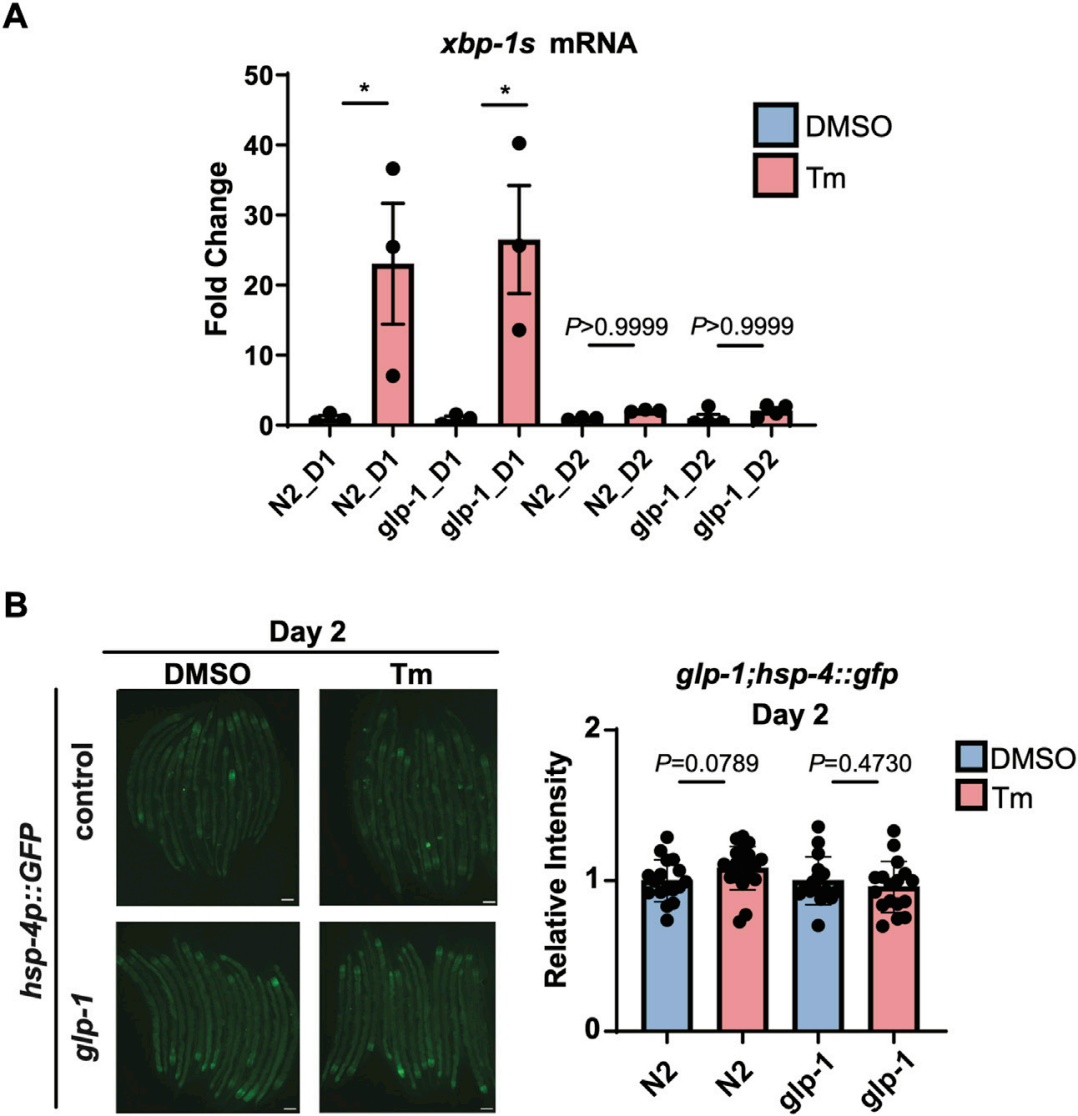
*glp-1* mutation does not prevent age-related decline in UPR^ER^ activation. **(A)** qRT-PCR measurement of *hsp-4* and *xbp-1s* mRNA after 6 h of tunicamycin (Tm) or DMSO treatment in N2 or *glp-1*(*e2141*) animals at the beginning of Day 1 (D1) or Day 2 (D2) of adulthood. *p < 0.05, ns = not significant, One-Way ANOVA with Tukey’s multiple comparison test. **(B)** Epifluorescence microscopy and quantification of the fluorescence of N2 or *glp-1* animals at Day 2 (D2) of adulthood after 6 h of treatment with Tunicamycin (Tm) or DMSO. Scalebars, 200 µm. Significance assessed by Unpaired *t*-test (DMSO vs. Tm).

We then turned our attention to whether this age-associated loss of IRE-1 activity can be prevented or reversed. It has been previously shown that *glp-1* mutant animals, which lack a functioning germline, are still capable of activating the HSR at day 2 of adulthood, and have generally higher levels of stress resistance (Labbadia and Morimoto, 2015a). We observed wild type levels of *xbp-1* splicing upon tunicamycin treatment in *glp-1*(*e2141*) animals at the beginning of day 1 of adulthood; however, by day 2, *xbp-1* splicing had been completely lost (Figure 4A). In addition, no activation of *hsp-4p::GFP* was seen in *glp-1*(*e2141*) animals at day 2 (Figure 4B). Furthermore, reducing levels of the germline-regulated H3K27 demethylase *jmjd-3.1* to mimic its decreased expression upon the onset of reproduction (Labbadia and Morimoto, 2015a), did not affect the ability of neuronally-overexpressed *xbp-1s* to regulate *hsp-4p::GFP* (Supplementary Figure S4). This suggests, surprisingly, that unlike other stress response pathways which also decline at the onset of the reproductive period, age-associated loss of UPR^ER^ activation cannot be rescued by eliminating the formation of the germline, and does not seem to depend upon changes in chromatin accessibility at target gene promoters.

Finally, we asked whether IRE-1 activation after day 1 of adulthood could be rescued by treatment with a drug, IXA4, shown to enhance activation of mammalian Ire1 (Grandjean et al., 2020). Treatment with 200 μM IXA4 at the L3/L4 larval stage induced a modest degree of UPR^ER^ activation (Supplementary Figure S5A). However, treatment at day 2 of adulthood did not activate the UPR^ER^ (Supplementary Figure S5B). We also treated animals with tunicamycin in addition to IXA4, to ask whether the drug could potentiate UPR^ER^ activation in response to ER stress. While there was a trend towards higher *hsp-4::GFP* activation in this case, the difference was not statistically significant (Supplementary Figure S5B). This suggests that IXA4 treatment cannot itself activate IRE-1 in aged animals, nor can it unambiguously reverse the age-associated loss of UPR^ER^ activation upon ER stress in this system. The loss of IRE-1 activation with age in *C. elegans* therefore remains refractory to our efforts to restore it.

## Discussion

These findings reveal that the ability to activate the UPR^ER^ is lost at the beginning of the reproductive period in *C. elegans*; that this arises from a failure to activate the regulatory enzyme IRE-1, leading to a loss of both *xbp-1* splicing and RIDD activity; and that this loss cannot be rescued by a failure to develop a germline. These results have several implications.

This work as well as previous studies shows that the onset of reproduction is a time of general stress response decline in *C. elegans*, suggesting that this period of early adulthood plays a key role in aging (Labbadia and Morimoto, 2015a). This is consistent with germline/soma tradeoff models that explain the evolution of aging by the diversion of resources from somatic maintenance in order to focus an organism’s efforts on the production of progeny (Maklakov and Immler, 2016). A corollary of this is that preventing reproduction can delay aging by allowing energy to be invested instead into the maintenance of the soma. However, we show here that loss of UPR^ER^ activation cannot be delayed by inhibiting the development of the germline, uncoupling the loss of somatic maintenance from investment in progeny production. It also demonstrates that the mechanistic change underlying UPR^ER^ decline cannot be rescued by the endocrine signals that arise from the proliferating germline stem cells in *glp-1* mutants, differentiating it from the HSR, activation of which is restored in *glp-1* animals (Arantes-Oliveira et al., 2003; Berman and Kenyon, 2006; Labbadia and Morimoto, 2015a).

The mechanistic basis for loss of UPR^ER^ activation in the somatic cells of *C. elegans* seems to lie upstream of the transcriptional activation of UPR^ER^ targets. This again differentiates it from the HSR, in which loss of chromatin accessibility at promoters of target genes explains the loss of HSR activation at the beginning of the reproductive period (Labbadia and Morimoto, 2015a). Changes in chromatin structure have also been proposed to explain the loss of UPR^ER^ activation in a human fibroblast model of aging (Sabath et al., 2020). In this human cell model, Xbp1 is still spliced following ER stress in aged cells, but downstream target genes are no longer upregulated. In *C. elegans*, however, XBP-1 target genes can still be regulated in aged animals when *xbp-1s* is overexpressed, suggesting that the promoters of these genes remain accessible, and therefore that age-associated failure to activate the UPR^ER^ in these animals involves an upstream event (Taylor and Dillin, 2013). Indeed, we show here that *xbp-1* splicing itself is lost in early adulthood, as is RIDD activity against *flp-6* (Figures 2B,C). Interestingly, this is the first description of a decline in RIDD activity during early adulthood; as RIDD is relevant to several aspects of mammalian physiology, including adaptive immunity, lipid metabolism, cellular differentiation, and insulin regulation (Han et al., 2009; Lee et al., 2011; So et al., 2012; Osorio et al., 2014; Wang et al., 2018), this loss of RIDD activity with age has significant implications for mammalian healthspan.

Together, these findings strongly suggest a failure in IRE-1 activation with age, which cannot be explained by loss of IRE-1 expression (Figure 3). This raises the possibility that the mechanisms underlying age-associated loss of UPR^ER^ activation might vary, either between species, or in dividing (e.g., fibroblasts) vs. non-dividing cells (e.g., somatic cells of *C. elegans*). This latter idea is supported by recent work showing that the induction of *Xbp1* splicing by tunicamycin exposure is partially compromised in the hippocampus of aged mice (Cabral-Miranda et al., 2020). However, the reasons for this variation are unclear, as is the mechanistic basis for loss of IRE-1 activation in *C. elegans* cells. In addition, how these different mechanisms that lead to the collapse of proteostasis pathways are orchestrated to occur during the same narrow window in early adulthood remains an open question. One possibility is that these events may share the same underlying molecular cause; for example, alterations in the redox environment in *C. elegans* can perturb both IRE-1 RNAse activity and histone methylation events (Hourihan et al., 2016; Bazopoulou et al., 2019).

Understanding this underlying mechanism would facilitate the design of therapeutics to reactivate Ire1, which may have applications in treating age-associated disease, including neurodegenerative disorders that are associated with dysregulated UPR^ER^ activation (Hetz and Mollereau, 2014). Neither strategy that we deployed to delay or restore UPR^ER^ activation in aged animals–germline inhibition or treatment with the Ire1-activating drug IXA4 (Grandjean et al., 2020)—significantly rescued UPR^ER^ activation in animals at day 2 of adulthood. As IXA4 was identified through screening based on activation of mammalian Ire1, which has notable differences from *C. elegans* IRE-1, it is possible that the drug is either not delivered to the relevant cells in our assays, or that it is not activating *C. elegans* IRE-1 as effectively as mammalian Ire1. Understanding the molecular basis for the inactivation of this enzyme would allow the design of more targeted approaches—for example, a failure in kinase domain activation would suggest the trial of specific kinase activating molecules.

Finally, a previous study showed that a mutation in *eat-2*, proposed to act as a mimetic of dietary restriction, is capable of partially rescuing the age-related decline in UPR^ER^ activation (Matai et al., 2019). This suggests that studying downstream effectors of the *eat-2* pathway, such as the transcription factors PHA-4 and SKN-1, might help us to understand the basis of age-related decline in IRE-1 activity and how it might be mitigated (Park et al., 2010; Glover-Cutter et al., 2013; Hourihan et al., 2016; Matai et al., 2019). Future work to identify the mechanism underlying molecular failure of IRE-1 in older cells, the mechanistic basis of UPR^ER^ rescue, and the relevance of these mechanisms in non-dividing human cells such as neurons, is likely to be of significant interest in tackling age-related disease.

## Data Availability

The original contributions presented in the study are included in the article/Supplementary Material, further inquiries can be directed to the corresponding author.
