# Supplementary figures and images for: IRE-1 endoribonuclease activity declines early in C. elegans adulthood and is not rescued by reduced reproduction
Source: Front Aging. 2022 Oct 28;3:1044556. doi: 10.3389/fragi.2022.1044556 (PMC9649906; doi:10.3389/fragi.2022.1044556)

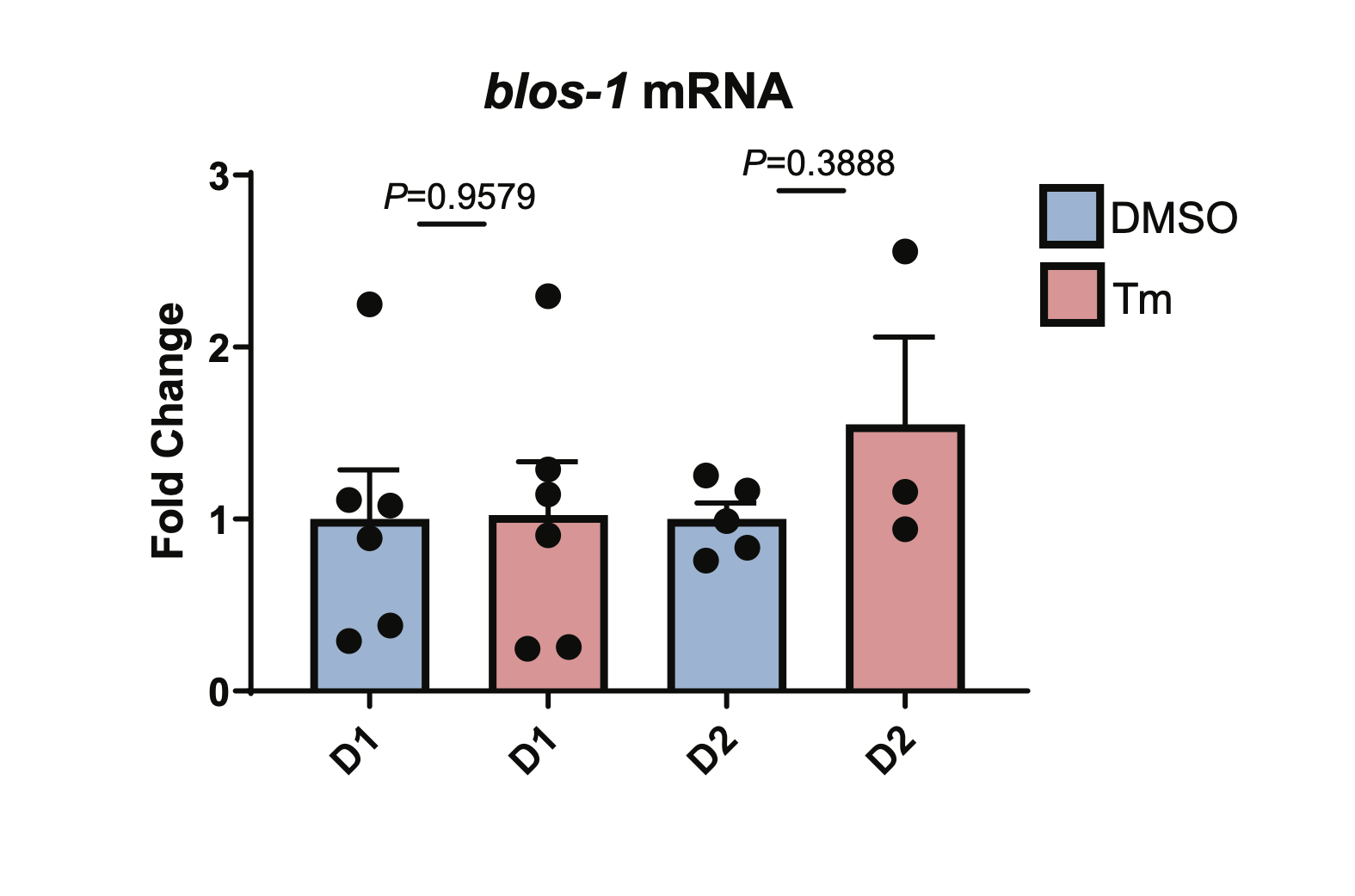

Supplement: Supplementary file 1 [file Image3.TIFF]

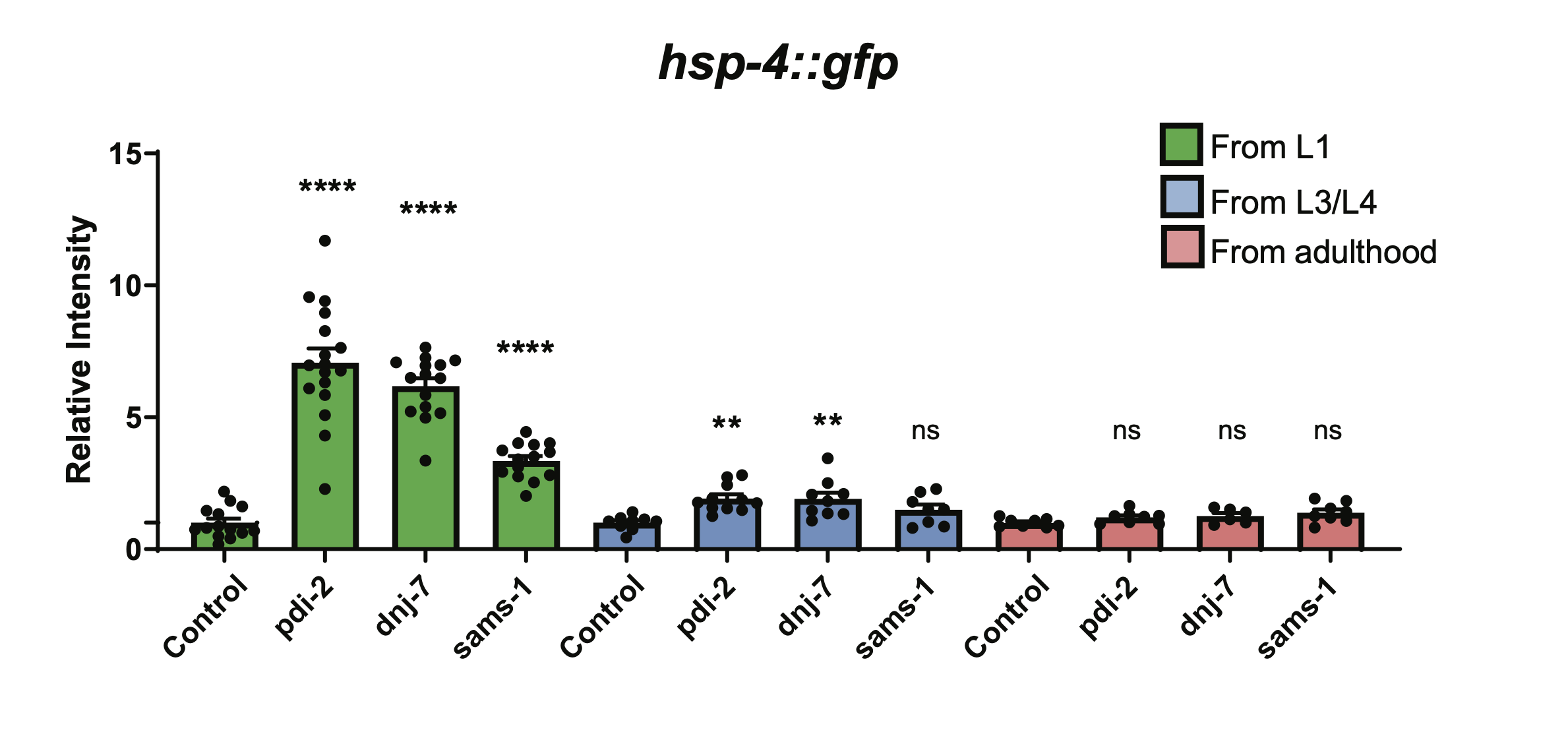

Supplement: Supplementary file 2 [file Image1.TIFF]

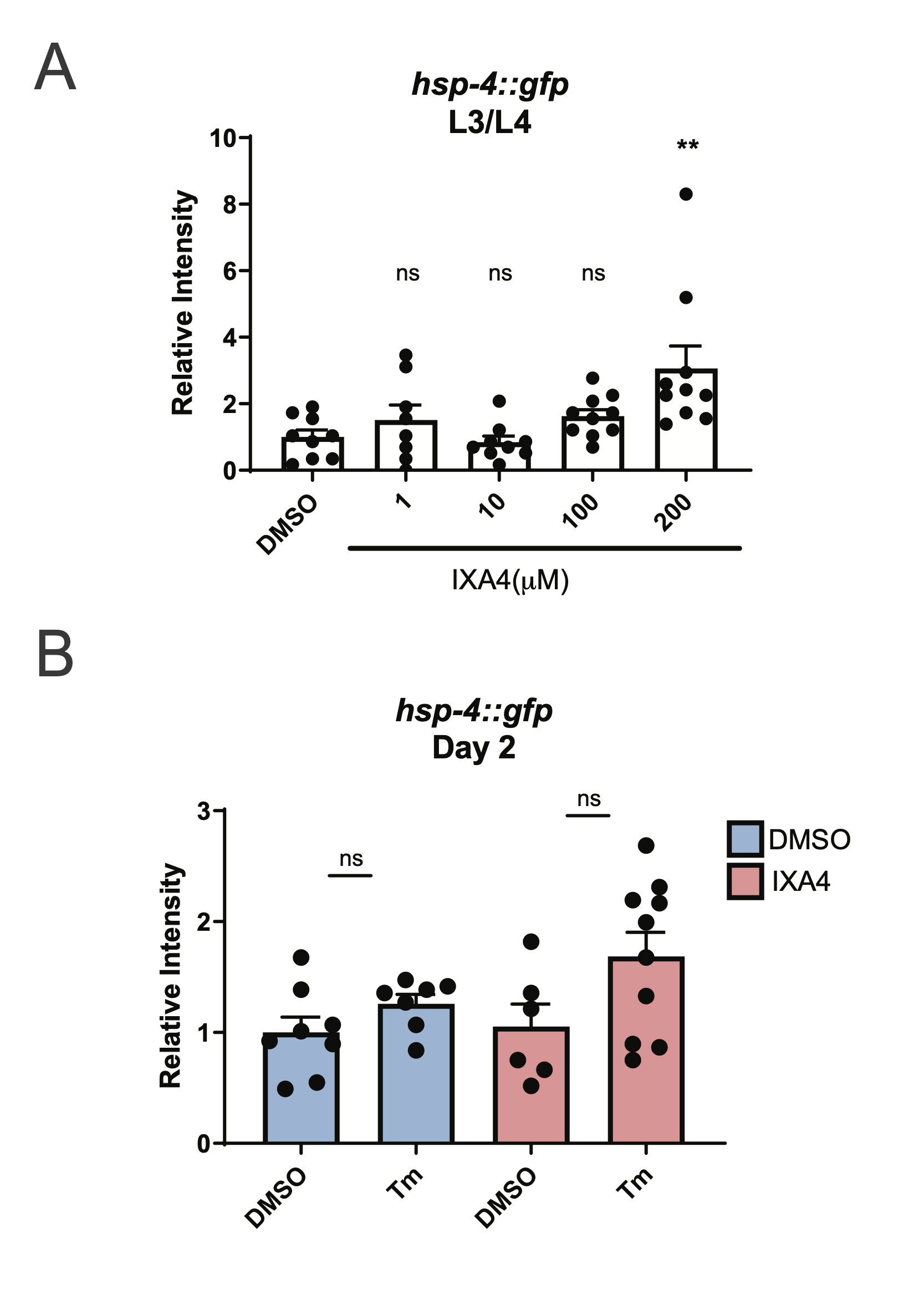

Supplement: Supplementary file 3 [file Image5.TIFF]

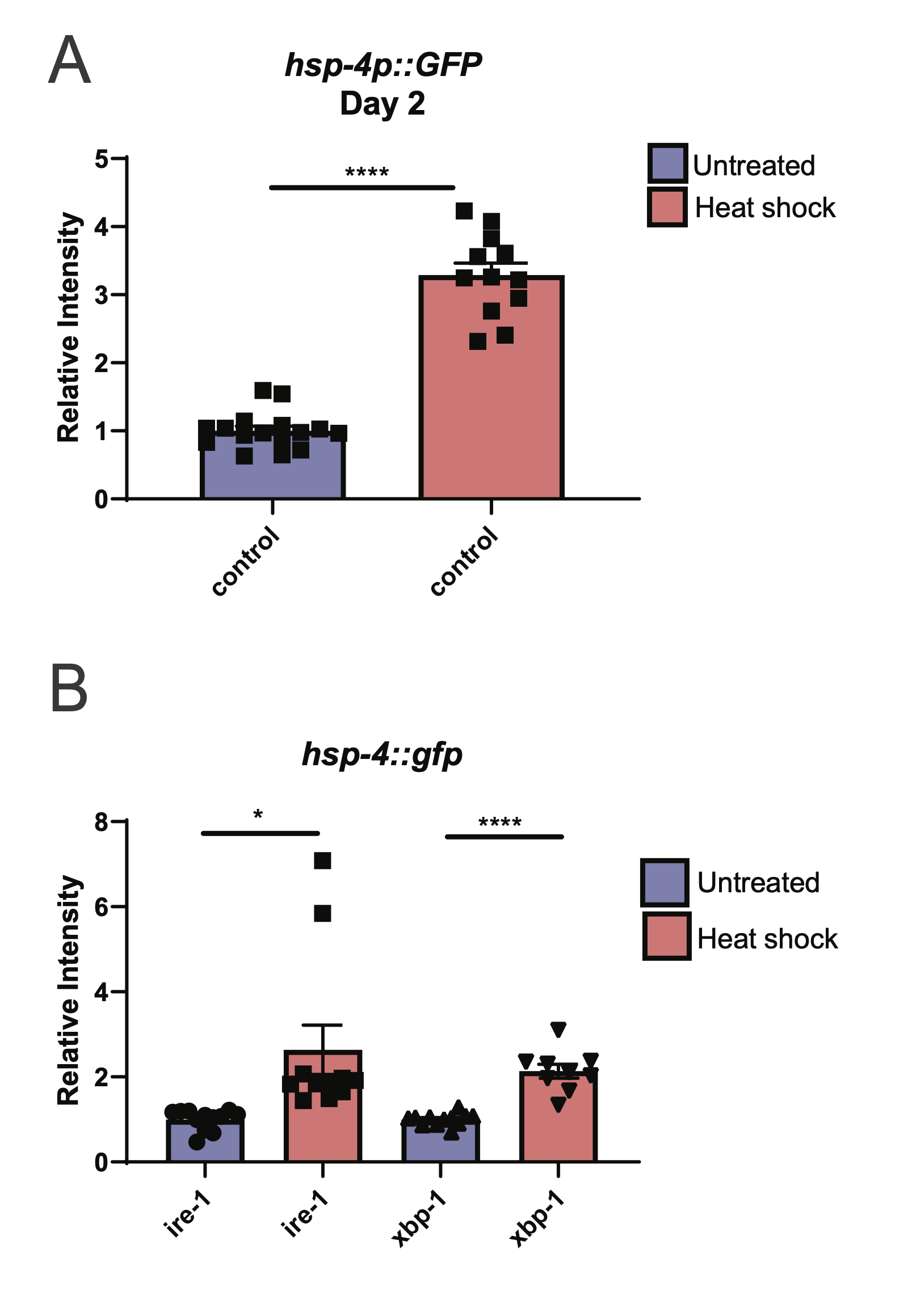

Supplement: Supplementary file 5 [file Image2.TIFF]

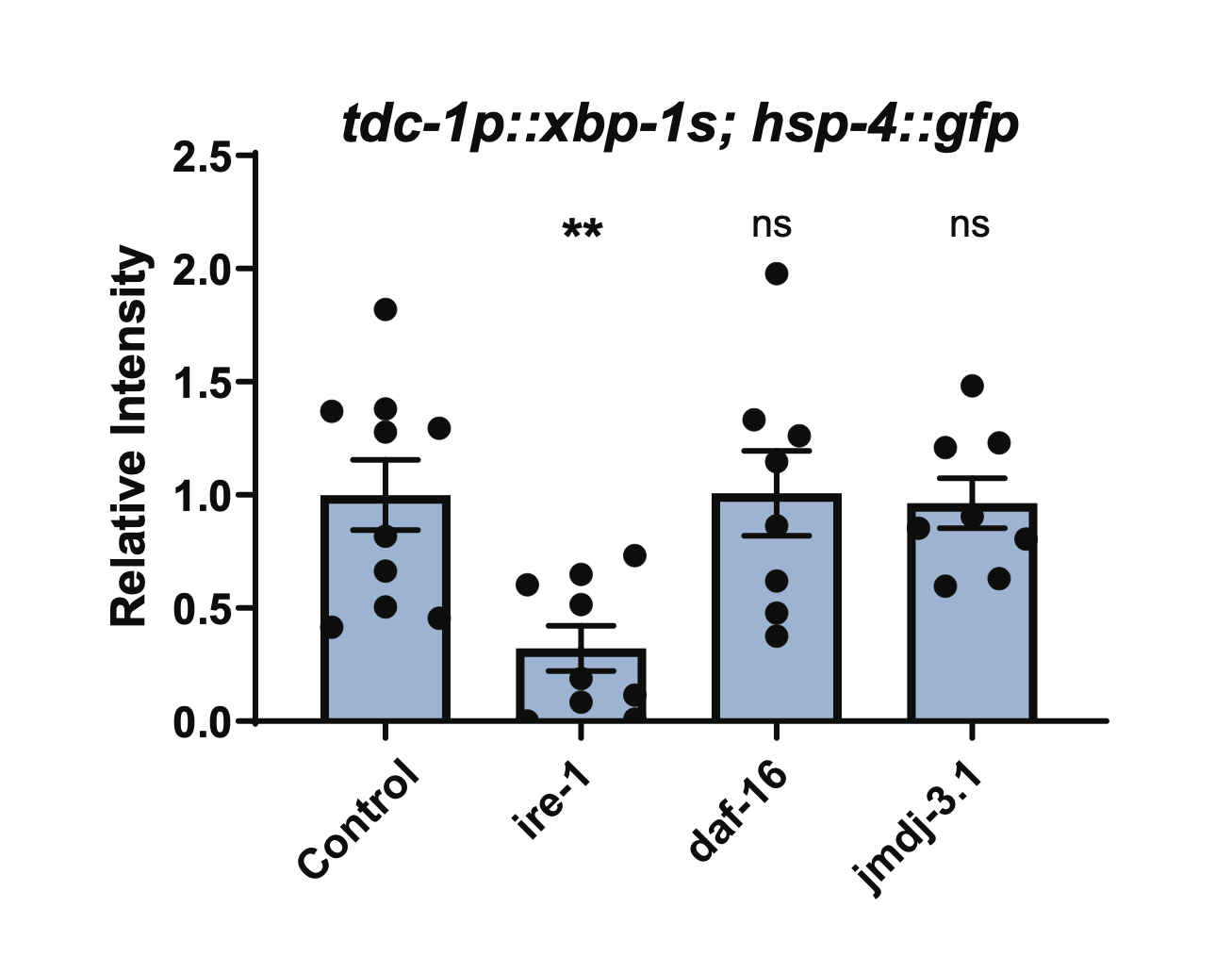

Supplement: Supplementary file 6 [file Image4.tiff]
