# Supplementary material for: IRE-1 endoribonuclease activity declines early in C. elegans adulthood and is not rescued by reduced reproduction
Source: Front Aging. 2022 Oct 28;3:1044556. doi: 10.3389/fragi.2022.1044556 (PMC9649906; doi:10.3389/fragi.2022.1044556)
Supplement: Supplementary file 4 [file DataSheet1.pdf]

# Supplementary Information

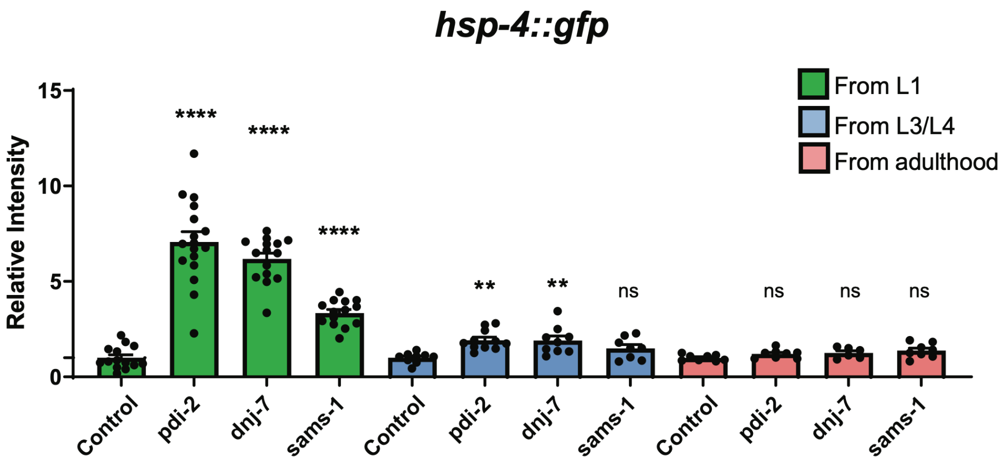


**Figure S1. Age-related decline in UPR$^{ER}$ activation is also observed when ER stress is induced using RNAi.** Animals at different stages (L1, L3/L4, and adulthood) were exposed to bacteria harboring RNAi against *pdi-2*, *dnj-7*, and *sams-1* to induce ER stress, or L4440 empty vector control, and the levels of *hsp-4p::GFP* were monitored after 72 hours of RNAi exposure by epifluorescence microscopy and ImageJ quantification. **** $P$<0.0001, **$P$<0.01, ns=not significant, One-Way ANOVA with Dunnett's multiple comparisons test.

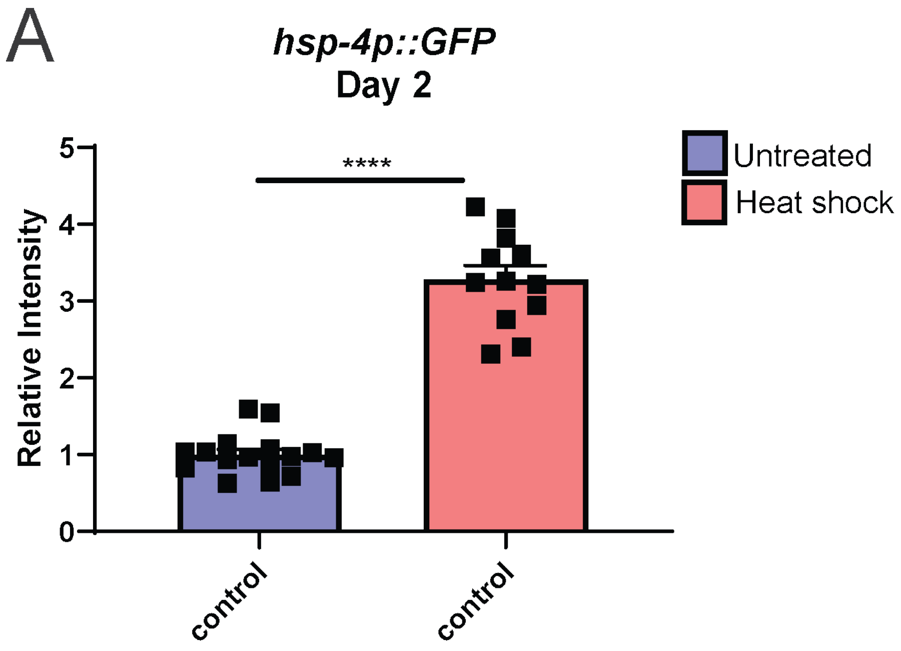


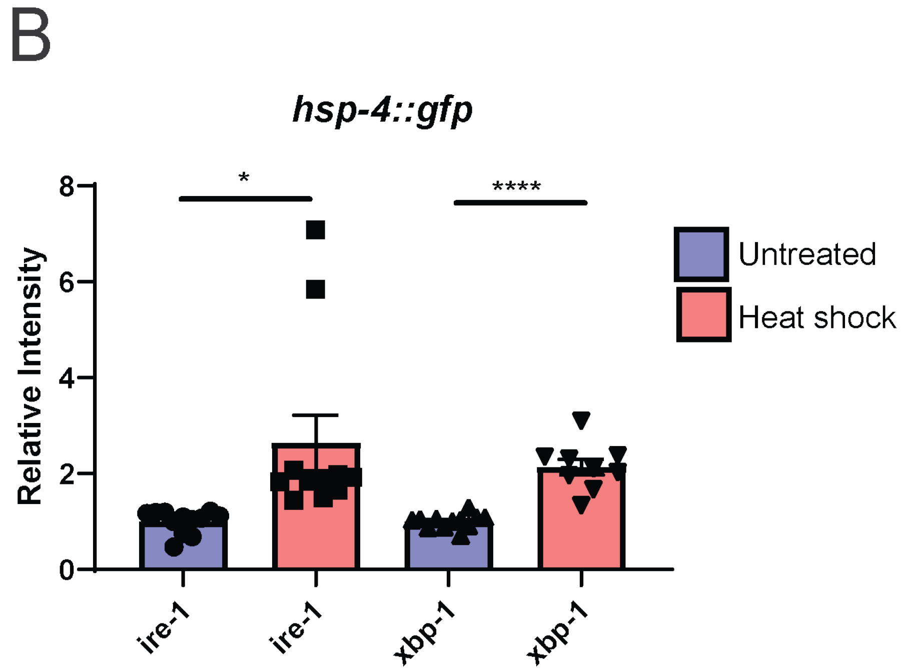


**Figure S2. Activation of *hsp-4p::GFP* by heat stress is not affected by the onset of the reproduction period and is not regulated by the IRE-1/XBP-1 pathway.** Animals expressing the *hsp-4p::GFP* transgene in a wild type (control) background were exposed to 4 hours of heat stress (34 °C) at Day 1 or Day 2 of adulthood, and were then imaged after 16 hours and their fluorescence quantified. ****$P<0.0001$, Unpaired t-test. **B.** *ire-1* and *xbp-1* mutants expressing the *hsp-4p::GFP* transgene were exposed to 4 hours of heat stress (34 °C) at Day 1 of adulthood and were imaged after 16 hours and their fluorescence quantified. ****$P<0.0001$, *$P<0.05$, Unpaired t-test (untreated vs heat stress).

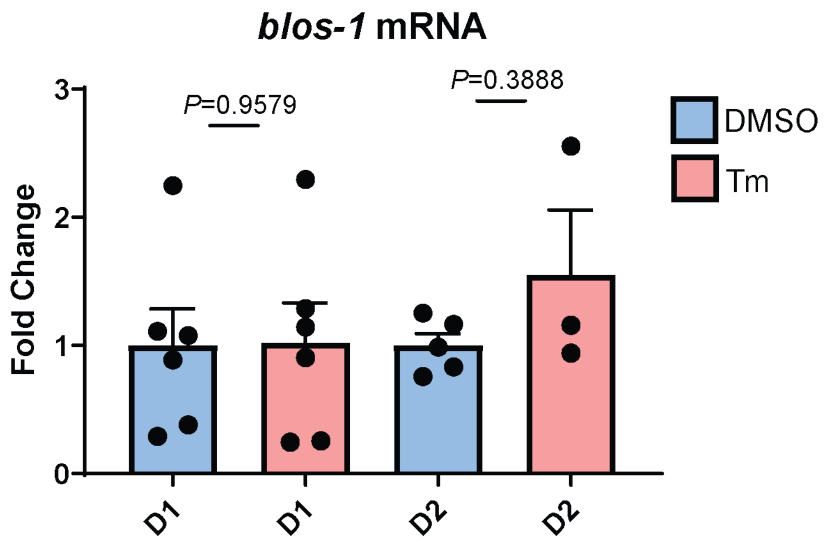


**Figure S3.** ***blos-1*** **mRNA levels are not affected by tunicamycin treatment in** ***C. elegans*****.** Animals at Day 1 or Day 2 of adulthood were exposed to tunicamycin (Tm) or DMSO for 6 hours and *blos-1* mRNA levels measured by qRT-PCR. Significance assessed by Unpaired t-test (DMSO vs Tm).

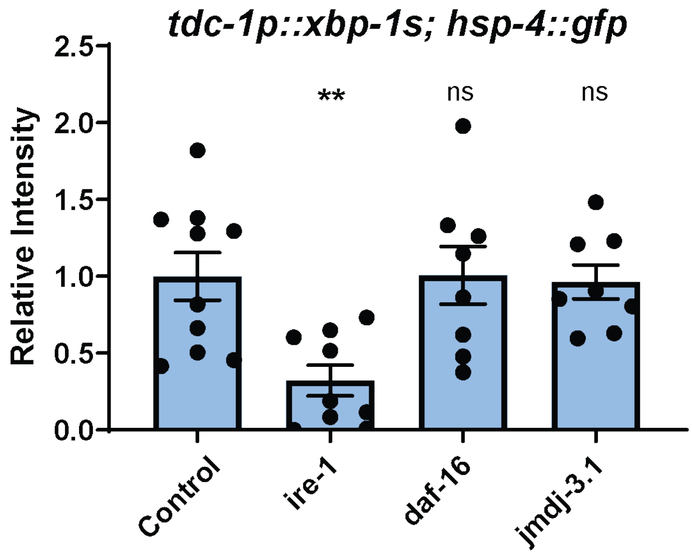


**Figure S4. DAF-16 and JMDJ-3.1 are not involved in the regulation of the UPR$^{ER}$ when *xbp-1s* is expressed in the RIM/RIC neurons.** *Tdc-1p::xbp-1s*; *hsp-4p::GFP* animals were grown on plates containing bacteria harboring different RNAis or L4440 empty vector control, imaged at Day 1 of adulthood, and their fluorescence quantified. **$P$<0.01, ns=not significant, One-Way ANOVA with Dunnett's multiple comparisons test.

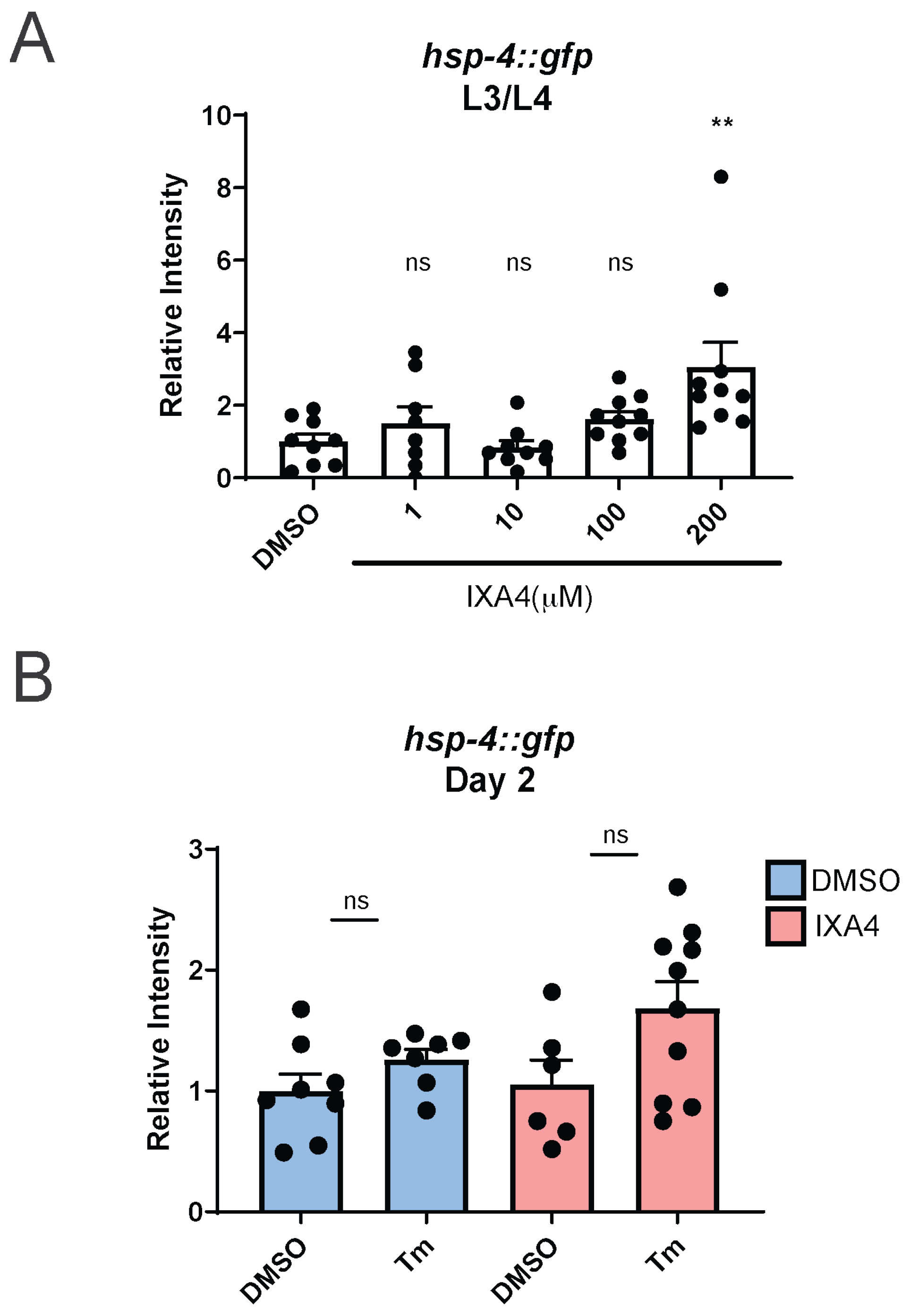


**Figure S5. IXA4 is capable of activating the UPR$^{ER}$ in *C. elegans*, but does not significantly prevent its decline with the onset of the reproductive period. A.** Animals expressing *hsp-4p::GFP* were treated for 6 hours with DMSO, tunicamycin (Tm), or different concentrations of IXA4 at the L3/L4 larval stage, imaged by epifluorescence microscopy, and fluorescence quantified by ImageJ. ***P*<0.01, ns=not significant, One-Way ANOVA with Dunnett's multiple comparisons test. **B.** Animals were grown on plates containing 200 μM of IXA4 or DMSO, and at Day 2 of adulthood were exposed to 6 hours of tunicamycin (Tm) or DMSO, imaged, and their fluorescence quantified. Significance assessed by Unpaired t-test (DMSO vs Tm).

**Table S1. List of *C. elegans* strains used in this work.**

| Name | Description |
|---|---|
| N2 | Wild-type, Bristol |
| SJ4005 | *zcIs4* [*hsp-4::GFP*] V |
| SJ30 | *ire-1(zc14)* II; *zcIs4* [*hsp-4::GFP*] V |
| AGD972 | *xbp-1(zc12)* III*; zcls4*[*hsp-4::GFP*] V |
| CB4037 | *glp-1*(*e2141*) III |
| RCT179 | *glp-1*(*e2141*) III; *zcIs4* [*hsp-4::GFP*] V |
| RCT191 | *rmsIs6 [tdc-1p::xbp-1s, cc::RFP]; zcIs4* [*hsp-4::GFP*] V |
| RCT208 | *ire-1(rms22[3xFLAG::wrmScarlet:ire-1])* II |

**Table S2. List of primers used in this work.**

| Name | Sequence (5' – 3') |
|---|---|
| *pmp-3* F | GTTCCCGTGTTCATCACTCAT |
| *pmp-3* R | ACACCGTCGAGAAGCTGTAGA |
| *cdc-42* F | CTGCTGGACAGGAAGATTACG |
| *cdc-42* R | CTCGGACATTCTCGAATGAAG |
| *Y45F10D.4* F | GTCGCTTCAAATCAGTTCAGC |
| *Y45F10D.4* R | GTTCTTGTCAAGTGATCCGACA |
| *ire-1* F | GGTGGATGGAGGGAGAAGATT |
| *ire-1* R | GAAGTAATAGCCGAGGGAAGC |
| *xbp-1_total* F | CACCTCCATCAACAACAACAT |
| *xbp-1_total* R | AACCGTCTGCTCCTTCCTCAA |
| *xbp-1_spliced* F | CGTGCCTTTGAATCAGCAGTG |
| *xbp-1_spliced* R | CGAGGTGTCCATCTTCTTGTT |
| *xbp-1_unspliced* F | AGAAGTCGTCGGTGAGGTTG |
| *xbp-1_unspliced* R | CCTGTTCCCACTGCTGAG |
| *xbp-1* splicing RT-PCR F | TCCGCTTGGGCTCTTGAGATGTTC |
| *xbp-1* splicing RT-PCR R | TGTCGTCGTCGGAGGAGAGGATCG |
| *flp-6* F | GTGAAGTGGAGAGAGAAATGATGA |
| *flp-6* R | CCGCTACTTCTCTTTCCAAAACG |
| *rtcb-1* F | GGAAGTCGAGGACTTGGACA |
| *rtcb-1* R | GTTAACCCAGGCGAAGTTTG |
